# Rheumatism and Its Successful Treatment

**Published:** 1895-12

**Authors:** William C. Wile

**Affiliations:** Danbury, Conn.


### Abstracts and Selections.

### Rheumatism and its Successful Treatment.

WILLIAM C. WILE, A. M., M. D., LL. D., DANBURY, CONN.

Rheumatism, under its many forms and guises, is one of the most common diseases that the New England doctor has to treat, and while the simpler forms yield readily to the proper medication, the majority are stubborn and resist all efforts of the practitioner to rout it. The following cases, successfully treated with tongaline, are, I believe, of sufficient interest to warrant their publication:

Carrie E., American, aged 9 years, was taken ill on the 17th day of January, and I was called to see her two days after.

I found the left knee and right ankle swollen, and so exquisitely painful to the touch, she would scream out on every attempt to move her in the bed. Pulse 120, tongue furred, and the temperature 102.2. I ordered the following:

℞ Hydrarg. chlor. mite.......................gr. v
Pulv. rhei...........................gr. ij
Sodo bicarb...........................gr. v

Sig. Make two powders. Give both at once and follow with a dose of castor oil in the morning.

℞ Tongaline............................℥ ij

Sig. Give thirty drops every hour until ten doses have been taken and then give every two hours.

The next morning I found that the calomel had acted freely, and as a consequence the temperature fell ½ a deg., the tongue looked better and the pulse was down to 100. There was no change, however, in the condition of the limbs. I was called out of town early the 21st and did not return until late on the 22nd. On my visit at that time I found the condition of the patient very materially improved. The swelling had diminished very considerably and the pain was much less. Had a fair night's rest the night before, the first she had had without an opiate since the commencement of her sickness. The tongaline was ordered given only every three hours, and her improvement was rapid and satisfactory. In two weeks' time she was about the house and all traces of the rheumatism gone. This case was similar to many others treated by other and improved methods that would have taken six weeks to cure. The diet had consisted entirely of bovinine and milk. There were no complications.

Mrs. C., aged fifty-nine, Irish, whose surroundings were of the worst, was taken with inflammatory rheumatism of the left knee joint.

It was swollen terribly, and the pain was so great that she got no rest. Four compound cathartic pills, U. S. P., were given and the alimentary canal thoroughly unloaded, when tongaline was given in teaspoonful doses every two hours. The pain began to be relieved in twenty-four hours, the swelling went down in forty-eight hours and she had entirely recovered in three weeks. No heart complications ensuing.

The next was one of those distressing cases of muscular rheumatism of the subacute variety, in a Mr. D., aged 44, American, laborer, who came to me suffering with great pains in the muscles of the arms and shoulder. The pain was such as to incapacitate him from pursuing his occupation as a track hand on the New York, New Haven & Hartford R. R. He commenced using tongaline in teaspoonful doses three times a day and at bed-time. The result was apparent by great improvement in two days and in one week's time the pain had all left him though I continued the administration of the medicine for a week longer in order to make assurance doubly sure. Since his first attack he had one other following a drenching in a rain, which occurred about three months after. This treatment brought about the same happy result.

I was called March 3, to see Mr. B., aged 37, Irish, laborer, who I found confined to his bed unable to move for severe pain in the lumbar region, affecting all the big muscles of that part.

It was impossible for him to turn over in bed or to help himself in any way. I ordered a big piece of flannel to be spread thickly with equal parts of ichthyol and wool-ola, and bound over the back tightly, giving two teaspoonfuls of tongaline every three hours night and day. Improvement took place in twelve hours, so that he could move his body with comparative ease, though a good deal of pain still existed. The improvement continued so rapidly that at the end of the third day he was sitting up, and the medicine was ordered to be taken in teaspoonful doses three times a day. A week of this completed the cure, and at the end of ten days he returned to his work on the streets where he had been employed before being taken ill.

These cases are of different types and illustrate the value of tongaline in all the different forms of the disease. I have a record of eighteen cases which have been treated with tongaline, and all of which were successful excepting only one, and that was complicated with gout.

